# Theoretical Study on Singlet Fission Dynamics and Triplet Migration Process in Symmetric Heterotrimer Models

**DOI:** 10.3390/molecules29225449

**Published:** 2024-11-19

**Authors:** Hajime Miyamoto, Kenji Okada, Kohei Tada, Ryohei Kishi, Yasutaka Kitagawa

**Affiliations:** 1 Graduate School of Engineering Science, Osaka University, Toyonaka 560-8531, Osaka, Japan; 2 Research Center for Solar Energy Chemistry (RCSEC), Graduate School of Engineering Science, Osaka University, Toyonaka 560-8531, Osaka, Japan; 3 Center for Quantum Information and Quantum Biology (QIQB), Osaka University, Toyonaka 560-8531, Osaka, Japan; 4 Innovative Catalysis Science Division, Institute for Open and Transdisciplinary Research Initiatives (ICS-OTRI), Osaka University, Suita 565-0871, Osaka, Japan; 5 Spintronics Research Network Division, Institute for Open and Transdisciplinary Research Initiatives (OTRI-Spin), Toyonaka 560-8531, Osaka, Japan

**Keywords:** singlet fission, exciton dynamics, hetero-fission, correlated triplet pair

## Abstract

Singlet fission (SF) is a photophysical process where one singlet exciton splits into two triplet excitons. To construct design guidelines for engineering directional triplet exciton migration, we investigated the SF dynamics in symmetric linear heterotrimer systems consisting of different unsubstituted or 6,13-disubstituted pentacene derivatives denoted as ***X/Y*** (***X***, ***Y***: terminal and center monomer species). Time-dependent density functional theory (TDDFT) calculations clarified that the induction effects of the substituents, represented as Hammett’s para-substitution coefficients $\sigma_{p}$, correlated with both the excitation energies of S_1_ and T_1_ states, in addition to the energies of the highest occupied and lowest unoccupied molecular orbitals (HOMO and LUMO). Electronic coupling calculations and quantum dynamics simulations revealed that the selectivity of spatially separated TT states for heterotrimers increased over 70%, superior to that in the homotrimer: an optimal region of the difference in $\sigma_{p}$ between the substituents of ***X*** and ***Y*** for the increase in SF rate was found. The origin of the rise in SF rate is explained by considering the quantum interference effect: reduction in structural symmetry opens new interaction paths, allowing the S_1_-TT mixing, which contributes to accelerating the hetero-fission between the terminal and center molecules.

## 1. Introduction

Singlet fission (SF) is a photophysical process where one singlet exciton (S_1_) is converted to two triplet excitons (2 × T_1_) via a correlated triplet pair (TT) [1,2]. SF is expected to improve the photoelectronic conversion efficiency of organic solar cells, overwhelming the Shockley–Queisser limit [3,4]. The SF process is divided into the initial TT generation and spin decoherence processes. For the initial process, it is convenient to classify the controlling factors of SF into three elements [5,6]: (i) energy level matching conditions presented by Michl et al. $\Delta E_{SF}=E_{S_{1}}-2E_{T_{1}\;}\;\geq \;0$, $\Delta E_{rec}=E(T_{2})-2E(T_{1})\geq 0$ [1,2], (ii) the electronic coupling referring to the electron and exciton transfer originated from π-orbital overlap between dye molecules [1,7,8], and (iii) exciton non-equilibrium dynamics driven by vibronic coupling (correlation between the exciton states and the vibrational phonon mode) [9,10,11,12]. Comprehensive design guidelines for SF materials can be obtained by organizing these factors.

Recently, the triplet dissociation process of SF has attracted the attention of many researchers because the TT states with several spin multiplicities (^1^TT, ^3^TT, and ^5^TT) as intermediate spin-entangled states of SF [13,14] are potentially utilized for applications to the quantum spin information technology for sensing [15] and quantum computing [16]. The applications of SF to these quantum spin technologies have still been limited because of the technical bottleneck in tracking the spatial distribution and operating the spin states of individual triplet excitons. A combination of the electron-spin-resonance (ESR) and the scanning tunneling microscopy (STM) technologies potentially enables us to observe and manipulate the local spin at the atomic and molecular levels, [17] although further development of experimental techniques is necessary. Especially, the individual operation of the two triplet spins is expected to be difficult when the positions of two triplets are close because the operation on one molecule affects another. Furthermore, it is generally impossible to detect where the triplet excitons migrate in a crystal or an aggregate system consisting of identical molecules. In this regard, tuning the structures of molecules and aggregates can be an alternative strategy for separating two triplet excitons while maintaining the correlation between them and controlling the spatial distributions of TT excitons suitable for quantum spin operations.

**Figure 1:**
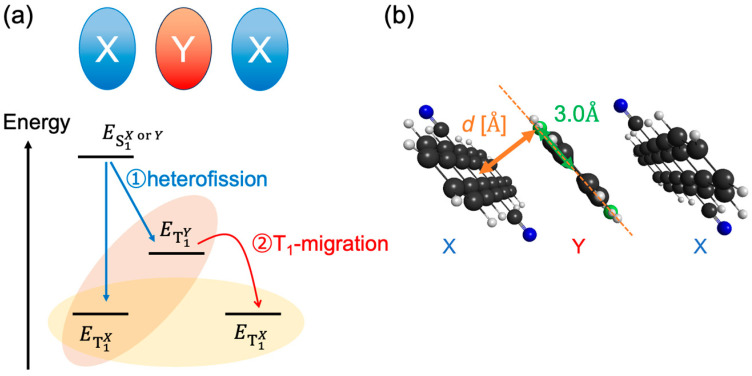
(**a**) The concept of a heterotrimer model consisting of molecules ***X*** and ***Y*** (denoted as ***X*/*Y***) and (**b**) the example of the structure of the slip-stacked trimer models of **CN2PEN/Cl2PEN** (N and Cl atoms are shown as blue and green, respectively) with the stacking distance *d* [Å] and fixed displacement along the short molecular axis by 3.0 Å.

In this study, we considered linear heterotrimer systems consisting of different pentacene derivatives, ***X*/*Y***, where ***X*** and ***Y*** denote the terminal and center monomer species (see Figure 1a) and propose design strategies for controlling the spatiotemporal distribution of TT states based on the results of quantum chemical calculations and dynamics simulation. With the heterotrimer model, we can examine the following effects: how the energy gradient of triplet excitation between ***X*** and ***Y*** affects the efficiency of directional triplet–triplet exciton transfer (TTET) from ***Y*** to ***X***, and how the exciton relaxation mechanism in heterotrimers differs from that in homotrimer. According to the previous research studies based on pentacene crystal structures [18,19,20,21,22] and ring-shaped aggregate structures [23,24], the SF rate decreased in highly symmetric systems because the interference effects tend to cancel the interaction paths between S_1_ and TT states mediated by various charge transfer (CT) states. This cancellation occurs when the excitons delocalize across entire aggregate systems and the energies of two CT states (the cation-anion state [CA] and the anion-cation state [AC]) are identical, which is applied to the ***X*** = ***Y*** case (homotrimer). When ***X*** ≠ ***Y*** (heterotrimer), the exciton fission is expected to occur between two different molecular species, which is called hetero-fission, and the energies of CA and AC states are not identical. These effects may resolve the cancellation of interaction paths and enhance the SF rate and TT yield.

This paper aims to clarify (i) the condition for ***X*** and ***Y*** optimizing both TT yield and the selectivity of the separated TT pair states on ***X*** molecules and (ii) the roles of asymmetric CT states for an efficient hetero-fission process. We considered a slip-stack configuration, as shown in Figure 1b, where *d* is the stacking distance. Although no covalent linkers directly connect the monomers, we expect that design strategies derived based on the present calculations can be applied to several covalently linked trimers like those synthesized by Nakamura et al. [25]. The present study is expected to help understand the mechanism and construct design guidelines of efficient SF materials that are useful for realizing the quantum spin manipulations for molecular materials, [26,27,28] although further discussions on the dynamics considering the high-spin TT pair states are necessary to establish comprehensive guidelines for efficient the quantum spin manipulations.

## 2. Methodology

### 2.1. Effective Energy-Matching Conditions for Heterotrimers

**Figure 2:**
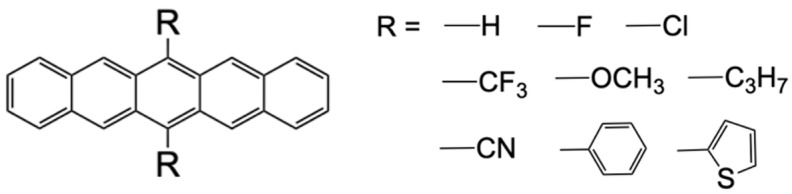
Molecular structures of unsubstituted pentacene **PEN** (R = H) and 6,13-disubstituted pentacene derivatives **R2PEN**.

For the monomers ***X*** and ***Y***, we considered unsubstituted and 6,13-disubstituted pentacenes (**PEN** and **R2PEN**, where R represents the substituent group shown in Figure 2). First, we conducted time-dependent density functional theory (TDDFT) calculation for the monomers only used in the pre-screening scheme for candidate systems. The geometrical optimization, TDDFT for vertical S_1_ excitation energy, and TDDFT with Tamm–Dancoff approximation (TDA) [29] for vertical T_1_ excitation energy of a single molecule were performed at the CAM-B3LYP/cc-pVDZ level [30] using Gaussian 16 package [31]. The monomer species examined in this study have been synthesized [32,33,34,35,36,37,38]. This pre-screening based on the TDDFT saves us the effort of calculating the electronic couplings of the dimer structure at the XMC-QDPT2/CASSCF(4,4) level for each ***X/Y*** pair.

Then, we considered effective energy-matching conditions for the heterotrimer case in terms of S_1_ ($E_{S_{1}^{X}},E_{S_{1}^{Y}}$) and T_1_ ($E_{T_{1}^{X}},\;E_{T_{1}^{Y}}$) energies of ***X*** and ***Y***, as follows:

(1) $$\Delta E_{SF}=\min\left(E_{S_{1}^{X}},E_{S_{1}^{Y}}\right)-2E_{T_{1}^{X}}\geq 0,$$

(2) $$\Delta E_{TTET}=E_{T_{1}^{X}}-E_{T_{1}^{Y}}<0,$$

The first equation is the isothermal/exothermal condition for hetero-fission, indicating that the energy of the final TT state should be lower than either the S_1_ energy of ***X*** or ***Y***. The second equation describes the exothermal condition for TTET from ***Y*** to ***X***.

### 2.2. Construction of Exciton Hamiltonian

We performed SF dynamics simulations for systems where the energy-matching conditions were satisfied appropriately. To construct the exciton Hamiltonian needed for the dynamics simulations, $H_{ex}$, we applied diabatic state approximation [1,2], including three S_1_ bases (S_1_S_0_S_0_, S_0_S_1_S_0_, and S_0_S_0_S_1_), four CT bases (CAS_0_, ACS_0_, S_0_CA, S_0_AC, where C and A denote the cation and anion, respectively), and three TT bases (TTS_0_, S_0_TT, and TS_0_T). Although the diabatic approximation is severely criticized due to its strong dependence on the construction method of the exciton model Hamiltonian [5] compared with the non-adiabatic coupling method [39,40], it is considered sufficient in the present study to obtain and prioritize concise physical insights of hetero-fission dynamics. The following 10 × 10 matrix expresses the exciton Hamiltonian in diabatic representation:

(3) $$H_{ex}=\left(\begin{array}{cccccccccc} E_{S_{1}^{X}} & V_{ex} & 0 & V_{S_{1}^{X}-C^{X}A^{Y}} & V_{S_{1}^{X}-C^{Y}A^{X}} & 0 & 0 & V_{S_{1}^{X}-T^{X}T^{Y}} & 0 & 0\\ & E_{S_{1}^{Y}} & V_{ex} & V_{S_{1}^{Y}-C^{X}A^{Y}} & V_{S_{1}^{Y}-C^{Y}A^{X}} & V_{S_{1}^{Y}-C^{Y}A^{X}} & V_{S_{1}^{Y}-C^{X}A^{Y}} & V_{S_{1}^{Y}-T^{X}T^{Y}} & V_{S_{1}^{Y}-T^{X}T^{Y}} & 0\\ & & E_{S_{1}^{X}} & 0 & 0 & V_{S_{1}^{X}-C^{Y}A^{X}} & V_{S_{1}^{X}-C^{X}A^{Y}} & 0 & V_{S_{1}^{X}-T^{X}T^{Y}} & 0\\ & & & E_{C^{X}A^{Y}} & 0 & 0 & 0 & V_{TT-C^{X}A^{Y}} & 0 & 0\\ & & & & E_{C^{Y}A^{X}} & 0 & 0 & V_{TT-C^{Y}A^{X}} & 0 & 0\\ & & & & & E_{C^{Y}A^{X}} & 0 & 0 & V_{TT-C^{Y}A^{X}} & 0\\ & & & & & & E_{C^{X}A^{Y}} & 0 & V_{TT-C^{X}A^{Y}} & 0\\ & & & & & & & E_{T^{X}T^{Y}} & 0 & t\\ & & & & & & & & E_{T^{X}T^{Y}} & t\\ & & & & & & & & & E_{T^{X}T^{X}} \end{array}\right)$$

The parameters $E_{S_{1}^{X}}$, $E_{S_{1}^{Y}}$ are the S_1_ excitation energies. The $E_{C^{X}A^{Y}}$ is defined as the energy of CT states for CAS_0_ and S_0_AC (cation is on one or another ***X*** and anion is on ***Y***), and $E_{C^{Y}A^{X}}$ is the opposite one indicating ACS_0_ and S_0_CA (cation is on ***Y*** and anion is on one or another ***X***). The $E_{T^{X}T^{Y}}$, and $E_{T^{X}T^{X}}$ are the TT state energies where the former indicates that (one of) ***X*** and ***Y*** are in their T_1_, the latter indicates that both ***X*** are in the T_1_. Therefore, if X = Y, $E_{C^{X}A^{Y}}=E_{A^{X}C^{Y}}$ and $E_{T^{X}T^{Y}}=E_{T^{X}T^{X}}$.

**Figure 3:**
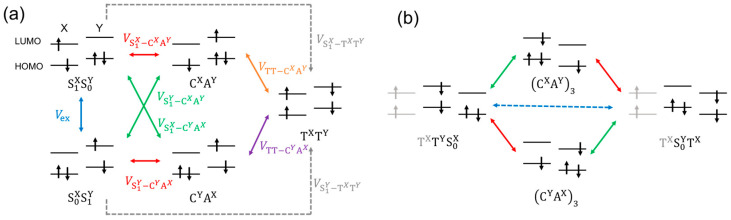
Schematic illustration of the diabatic CSFs and electronic coupling for (**a**) initial TT generation process of SF and (**b**) triplet migration process via virtual intermediate CT states. Double-headed arrows represent the transition paths.

The parameters appearing in the off-diagonal elements, $V_{ex}$, $V_{S_{1}^{X}-C^{X}A^{Y}}$, $V_{S_{1}^{Y}-C^{Y}A^{X}}$, $V_{S_{1}^{Y}-C^{Y}A^{X}}$, $V_{S_{1}^{X}-C^{Y}A^{X}}$, $V_{S_{1}^{X}-T^{X}T^{Y}}$, $V_{S_{1}^{Y}-T^{X}T^{Y}}$, $V_{TT-C^{X}A^{Y}}$, $V_{TT-C^{Y}A^{X}}$, and t are interpreted as follows:

1. $V_{ex}\sim \langle h_{1}l_{1}|h_{2}l_{2}\rangle$ (where the two-electron integral is defined by physicists’ representation: $\left\langle pq|rs\right\rangle =\int \varphi_{p}\left(1\right)\varphi_{q}\left(1\right)r_{12}^{-1}\varphi_{r}\left(2\right)\varphi_{s}\left(2\right)d^{3}r_{1}d^{3}r_{2}$) is the transfer integral of S_1_ exciton between neighboring monomers (FE coupling).
2. $V_{S_{1}^{X}-C^{X}A^{Y}}$, $V_{S_{1}^{Y}-C^{Y}A^{X}}$, $V_{S_{1}^{Y}-C^{Y}A^{X}}$, $V_{S_{1}^{X}-C^{Y}A^{X}}$ are CT-FE couplings. They are approximately interpreted as the Fock matrix elements denoted as LUMO-LUMO or HOMO-HOMO electron transfer integral $V_{LL}=\langle l_{X}\left|F\right|l_{Y}\rangle$ for $V_{S_{1}^{X}-C^{X}A^{Y}}$, $V_{S_{1}^{Y}-C^{Y}A^{X}}$ and $V_{HH}=\langle h_{X}\left|F\right|h_{Y}\rangle$ for $V_{S_{1}^{Y}-C^{Y}A^{X}}$, $V_{S_{1}^{X}-C^{Y}A^{X}}$, where the $l_{X}$, $l_{Y}$, $h_{X}$, and $h_{Y}$ represents the diabatic molecular orbitals localized to the lowest unoccupied molecular orbitals (LUMO) and the highest-occupied molecular orbitals (HOMO). If X = Y, $V_{S_{1}^{X}-C^{X}A^{Y}}=V_{S_{1}^{Y}-C^{Y}A^{X}}\sim V_{LL}$ and $V_{S_{1}^{Y}-C^{Y}A^{X}}=V_{S_{1}^{X}-C^{Y}A^{X}}\sim -V_{HH}$ are derived.
3. $V_{TT-C^{X}A^{Y}}$ and $V_{TT-C^{Y}A^{X}}$ are CT-TT couplings, which is dominantly represented as the Fock matrix element of LUMO and HOMO: $V_{TT-C^{X}A^{Y}}\sim \sqrt{3/2}V_{LH}=\langle l_{X}\left|F\right|h_{Y}\rangle$ and $V_{TT-C^{Y}A^{X}}\sim \sqrt{3/2}V_{HL}=\langle h_{X}\left|F\right|l_{Y}\rangle$. If X = Y, $\left|V_{TT-C^{X}A^{Y}}|=|V_{TT-C^{Y}A^{X}}\right|$.
4. $V_{S_{1}^{X}-T^{X}T^{Y}}$ and $V_{S_{1}^{Y}-T^{X}T^{Y}}$ are direct couplings between FE-TT states. Because these couplings only include two-electron integrals smaller than 0.1 meV, the effect of these couplings on SF dynamics is not significant compared with the CT-mediate coupling in this study.
5. t is the TTET coupling. In this study, we approximate this coupling by effective triplet transfer coupling obtained from quantum chemical calculation in dimer structure [41,42]. In this scheme, the perturbative ^3^CT-mediated triplet transfer pass shown in Figure 3b is added to the direct two-integral coupling as described in the following formula:
  (4a) $$t\equiv \left\langle T_{1}S_{0}|H|S_{0}T_{1}\right\rangle +\frac{\left\langle T_{1}S_{0}|H|{\left(C^{X}A^{Y}\right)}_{3}\right\rangle \left\langle {\left(C^{X}A^{Y}\right)}_{3}|H|S_{0}T_{1}\right\rangle }{\Delta E_{C^{X}A^{Y}}}+\frac{\left\langle T_{1}S_{0}|H|{\left(C^{Y}A^{X}\right)}_{3}\right\rangle \left\langle {\left(C^{Y}A^{X}\right)}_{3}|H|S_{0}T_{1}\right\rangle }{\Delta E_{C^{Y}A^{X}}},$$
  (4b) $$\frac{1}{\Delta E_{C^{X}A^{Y}}}=\frac{1}{2}(\frac{1}{E_{{{(C}^{X}A^{Y})}_{3}\;}-E_{T_{1}S_{0}}}+\frac{1}{E_{{{(C}^{X}A^{Y})}_{3}}-E_{S_{0}T_{1}}})$$
  (4c) $$\frac{1}{\Delta E_{C^{Y}A^{Y}}}=\frac{1}{2}(\frac{1}{E_{{{(C}^{Y}A^{X})}_{3}}-E_{T_{1}S_{0}}}+\frac{1}{E_{{{(C}^{Y}A^{X})}_{3}}-E_{S_{0}T_{1}}})$$

Each coupling parameter is obtained at the XMC-QDPT2/CASSCF(4e,4o)/6-31G(d) [43] level with the Nakamura–Truhlar 4-fold way diabatization scheme [44]. The diabatization and XMC-QDPT2 calculation were performed by GAMESS [45]. After obtaining the eigenvalues of excited states as the linear combination of configuration state functions (CSFs) represented by diabatic molecular orbitals (DMOs), the diabatic state energies and couplings are obtained by the unitary transformation of the diagonal Hamiltonian to the CSFs representation, which is proposed by Ma et al. [46]. The multi-excitonic and doubly excited states in the dimer structure are explicitly considered in this model through the CSFs for CAS(4e,4o) space. Details of the electronic couplings are given in the Supplementary Materials.

### 2.3. Quantum Master Equation

To describe the exciton relaxation dynamics, including state-to-state transitions, the interaction between nuclear vibrational mode (phonon mode) and exciton states, called vibronic coupling (VC), [7,9,47] should be considered. In this study, the time evolution of the exciton population was simulated using the time-convolutionless second-order quantum master equation (TCL-QME) within the Markov approximation, where we considered Holstein coupling [48]. The TCL-QME is a differential equation in time of reduced density matrix (RDM) $\rho_{ex}$ projected on the exciton states denoted as [49], as follows:

(5) $$\frac{d}{dt}\rho_{ex}=-i\left[H_{ex},\rho_{ex}\right]+\sum_{\omega ,m}\gamma \left(\omega \right)\left[A_{m}\left(\omega \right)\rho_{ex}\left(t\right)A_{m}{\left(\omega \right)}^{+}-\frac{1}{2}\left\{A_{m}\left(\omega \right)A_{m}{\left(\omega \right)}^{+},\rho_{ex}\left(t\right)\right\}\right]$$

where the function $\gamma \left(\omega \right)$ represents the relaxation rate, and the operator $A_{m}\left(\omega \right)=\sum_{\omega }\delta \left(\omega -E_{\alpha }+E_{\beta }\right)\left|\alpha \right\rangle \left\langle \alpha \right|m\rangle \left\langle m|\beta \right\rangle \langle \beta |$ is a quantum jump operator between adiabatic states α and β via the virtual intermediate diabatic exciton states m. The relaxation rate under Markov approximation is written as

(6) $$\gamma_{m}\left(\omega \right)=\left\{\begin{array}{c} 2\pi J_{m}\left(\omega \right)\left\{1+n_{B}\left(\omega ,T\right)\right\}\;\;\;\;\;\;\mathrm{for}\;\omega >0\\ 2\pi J_{m}\left(-\omega \right)n_{B}\left(-\omega ,T\right)\;\;\;\;\;\;\;\;\;\;\;\mathrm{for}\;\omega <0\\ \frac{4\lambda_{m}k_{B}T}{\Omega_{c}}\;\;\;\;\;\;\;\;\;\;\;\;\;\;\;\;\;\;\;\;\;\;\;\;\;\;\;\;\;\;\;\;\;\;\;\mathrm{for}\;\omega =0 \end{array}\right.,$$

where $J_{m}\left(\omega \right)$, $n_{B}\left(\omega ,T\right)$ is the spectral density and the Bose-Einstein distribution function at temperature T=300 K. At this temperature, we can assume that the phonon modes interact with the system as the bath. We should note that the vibrational coherence effects for SF, which is important at a low temperature and short-time region after the photoexcitation, can be treated by using a very sophisticated multi-configurational time-dependent Hartree (MCTDH) method. [50] The spectral density, which represents the effect of VC strength at a frequency ranging from ω to ω+dω, is modeled as an Ohmic function with a Lorentz–Drude cutoff, which is usually used to describe the system-bath correlation crucial for the SF relaxation dynamics [47,48], as follows:

(7) $$J_{m}\left(\omega \right)\equiv \frac{1}{\pi }\frac{2\lambda_{m}\Omega_{m}\omega }{\omega^{2}+\Omega_{m}^{2}}$$

where $\lambda_{m}$ is reorganization energy and $\Omega_{m}$ is a cutoff frequency. This spectral density indicates that the vibronic coupling distribution has a peak value of $\lambda_{m}/\pi$ at $\omega =\Omega_{m}$. In this study, we considered an identical spectral density case for different diabatic states ($\lambda =\lambda_{m}=50\;meV$ and $\Omega =\Omega_{m}=180\;meV$ [47]) and different molecular species, although we should note that considering the state-dependent reorganization energy, especially for the CT states, may potentially affect the TT generation rate and TT populations quantitatively. In addition, these VC parameters generally depend on the molecular species, but we focused only on the qualitative changes in the exciton Hamiltonian for simplicity. The Peierls coupling, which is the VC on the off-diagonal exciton Hamiltonian, was ignored in this study although they are expected to exhibit a peak at the low vibrational frequency mode. This is because the reorganization energy of Peierls coupling is generally much smaller than that of Holstein coupling, according to the previous study on SF dynamics of acene crystals [51]. The initial population was 100% for the S_1_ state on the center molecule ***Y***.

## 3. Results

### 3.1. Pre-Screening of Candidate Systems

**Figure 4:**
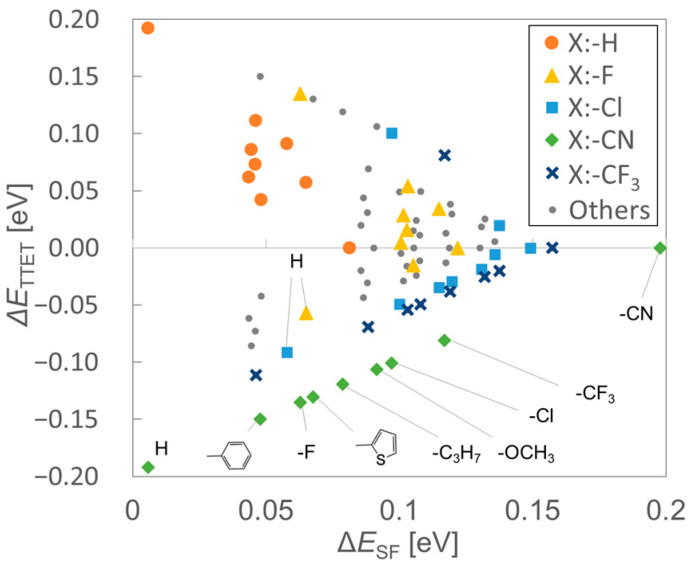
The SF exothermal condition ($\Delta E_{SF}=\min\left(E_{S_{1}^{X}},E_{S_{1}^{Y}}\right)-2E_{T_{1}^{X}}$) on the horizontal axis and the exothermic condition for triplet–triplet exciton transfer (TTET) from ***Y*** to ***X*** ($\Delta E_{TTET}=E_{T_{1}^{X}}-E_{T_{1}^{Y}}$) on the vertical axis in the pair of pentacene derivatives. The colored dots represent the data of fixed X = **R2PEN** for R = H (orange), F (yellow), Cl (light blue), CN (green), and CF_3_ (dark blue). The small gray dots denote the data including X = **OCH_3_2PEN**: R = CH_3_O, **Pr2PEN**: R = C_3_H_7_, **Ar2PEN**: R = C_6_H_5_, **Thi2PEN**: R = C_4_H_3_S.

First, we performed TDDFT calculations for monomer species to evaluate the energy-matching conditions, Equations (1) and (2), for pairs of ***X*** and ***Y*** shown in Figure 2. Figure 4 shows the plots of $\Delta E_{SF}$ (horizontal axis) vs. $\Delta E_{TTET}$ (vertical axis). The plots on the lower-right region indicate that both the yield of the TT state and the selectivity of the separated TT state (TS_0_T) are expected to be high. In Figure 4, the plots with the same marker indicate the results of ***X/Y*** with the same ***X***. From the results, the $E_{T_{1}^{X}}-E_{T_{1}^{Y}}$ become large negative in **CN2PEN/*Y*** system (green rhombus), where **CN2PEN** denotes 6,13-dicyanopentacene.

**Figure 5:**
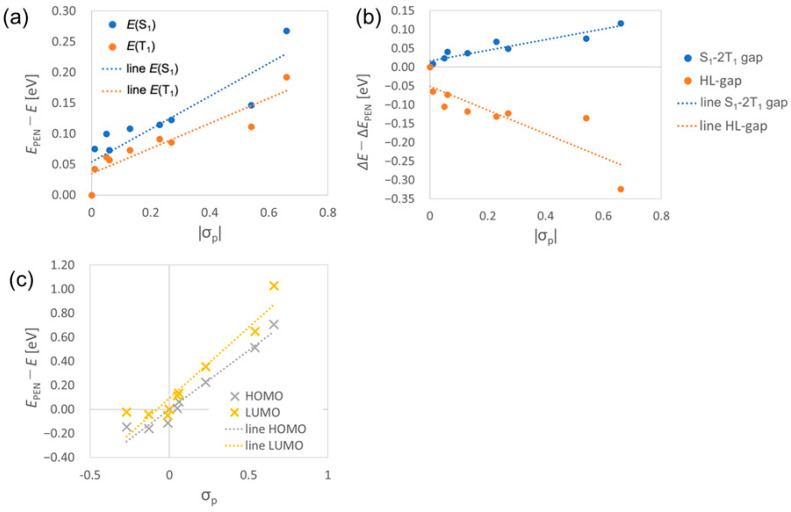
The correlations between (**a**) $\left|\sigma_{p}\right|$ and the excitation energies of the S_1_ and T_1_ states, (**b**) $\left|\sigma_{p}\right|$ and S_1_-2T_1_ energy gap and HOMO-LUMO gap, and (**c**) $\sigma_{p}$ and HOMO and LUMO energies. We took the corresponding energies or energy gaps of **PEN** (*E*_PEN_ or Δ*E*_PEN_) as the standards, and we plotted *E*_PEN_ − *E* in (**a**,**c**), whereas Δ*E* − Δ*E*_PEN_ in (**b**) for clarity. The dotted approximate line shows the general trends of $\sigma_{p}$ and several energies.

Figure 5a,b show the correlations between the absolute value of Hammett’s *para*-substitution constant, $\left|\sigma_{p}\right|$, [52] with the S_1_ and T_1_ excitation energies, and the S_1_-2T_1_ and HOMO-LUMO energy gaps, respectively, of pentacene derivatives ($\sigma_{p}$ value of each molecule is summarized in Appendix A). This result indicates that the SF process in homodimers of the pentacene derivatives tends to become exothermic as the $\left|\sigma_{p}\right|$ increases. The results also suggest that the inductive effects of the substituents can play a crucial role in tuning the excitation energies of heterotrimers. For example, $\Delta E_{SF}$ of **CN2PEN/F2PEN** (**2FPEN**: 6,13-difluoropentacene) was a significant positive value (0.063 eV), because both **CN2PEN** and **F2PEN** have large S_1_-2T_1_ gap, whereas that of **CN2PEN/PEN** (**PEN**: unsubstituted pentacene) was close to zero (0.006 eV). In contrast, the difference in the triplet energies between ***X*** and ***Y***, $\Delta E_{TTET},$ was smaller in **CN2PEN/F2PEN** than **CN2PEN/PEN** because of the smaller difference in $\left|\sigma_{p}\right|$ in the former. Additionally, the energies of HOMO and LUMO showed negative correlations with $\sigma_{p}$, as shown in Figure 5c (where the vertical axis shows the difference from that of pentacene: $E_{PEN}-E$). The relative energies of HOMO and LUMO between the different molecules affect the charge transfer character between the molecules.

### 3.2. Electronic Coupling

**Figure 6:**
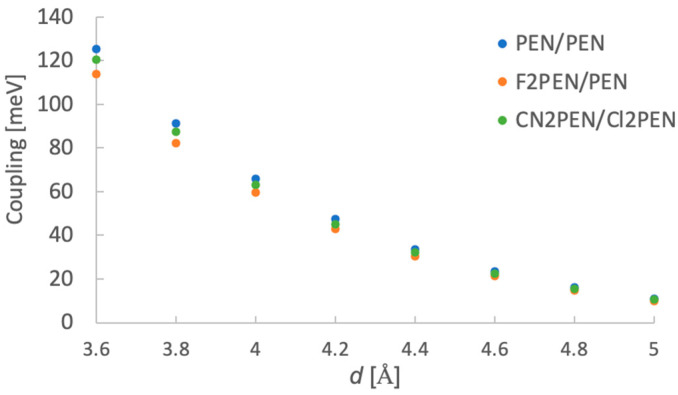
Calculation results of $V_{C^{Y}A^{X}-TT}$ as a function of stacking distance *d* [Å].

**Figure 7:**
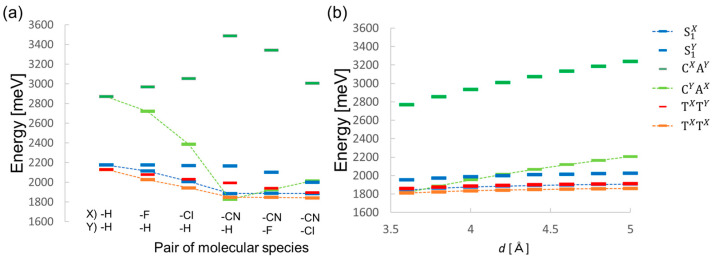
Calculation results of diabatic state energies (**a**) of different combinations ***X*/*Y*** and (**b**) of **CN2PEN/Cl2PEN** with different *d* [Å].

Next, we examined the diabatic energies and electronic couplings. The coupling parameters for the slip-stacked structure were evaluated, as shown in Figure 1b. The stacking distance *d* [Å] varied from 3.6 to 5.0 Å with an increment of 0.2 Å, and the displacement along the short molecular axis was fixed to 3.0 Å. In Figure 1b, we show an example of **CN2PEN/Cl2PEN** (**Cl2PEN**: 6,13-dichloropentacene). Figure 6 shows the calculation results of $V_{C^{X}A^{Y}-TT}$ coupling, presented in the off-diagonal elements of exciton Hamiltonian, as a function of *d* for **PEN/PEN**, **F2PEN/PEN**, and **CN2PEN/Cl2PEN**. These systems showed similar *d*-dependences of $V_{C^{X}A^{Y}-TT}$. It is clarified that the trends of the other off-diagonal electronic couplings also did not show apparent dependence on the molecular species of ***X*** and ***Y*** (see Supplementary Materials). In contrast, the diabatic state energies, especially the CT energies, strongly depend on ***X*** and ***Y*** molecular species. Figure 7a shows diabatic state energies of S_1_*^X^*, S_1_*^Y^*, C*^X^*A*^Y^*, C*^Y^*A*^X^*, T*^X^*T*^Y^*, and T*^X^*T*^X^* states for ***X/Y*** = **PEN/PEN**, **F2PEN/PEN**, **Cl2PEN/PEN**, **CN2PEN/PEN**, **CN2PEN/F2PEN**, and **CN2PEN/Cl2PEN**. Note that the diabatic state energies of S_1_*^X^* and T*^X^*T*^X^* were independent of the specie ***Y*** and that of S_1_*^Y^* was independent of the specie ***X***. As shown in Figure 5c, the energies of HOMO and LUMO lowered when for systems with strong electron-withdrawing groups (EWGs). Thus, when the $\sigma_{p}$ of ***X*** is larger than ***Y***, the energy of the C^Y^A*^X^* state tends to be stabilized. For example, the C*^Y^*A*^X^* state (light green) is lower in energy than S_1_*^X^* or T*^X^*T*^X^* states when ***X*** = **CN2PEN**, ***Y*** = **PEN**, and **F2PEN** for d≤ 3.8 Å, which may lead to lower TT yields in these models. On the contrary, the energy of C*^X^*A*^Y^* state (green) was high. However, as shown in Figure 7b which provides the *d*-dependence of diabatic state energies in **CN2PEN/Cl2PEN**, both the C*^Y^*A*^X^* and C*^X^*A*^Y^* state energies became higher as the *d* increased whereas S_1_ and TT diabatic state energies remained unchanged. Then, the C*^Y^*A*^X^* diabatic state energy became higher than the S_1_ and TT diabatic states as the *d* increased. Consequently, the low yields of TT owing to the overstabilization of the C*^Y^*A*^X^* state in d≤ 3.8 Å can be improved by increasing *d*.

Consequently, the CT energies are controllable by choosing appropriate ***X*** and ***Y*** molecular species and stacking distance *d*. The stabilization energies of CT states depend on the difference in the inducing effect of the substituents between ***X*** and ***Y***.

### 3.3. SF Dynamics Simulations

**Figure 8:**
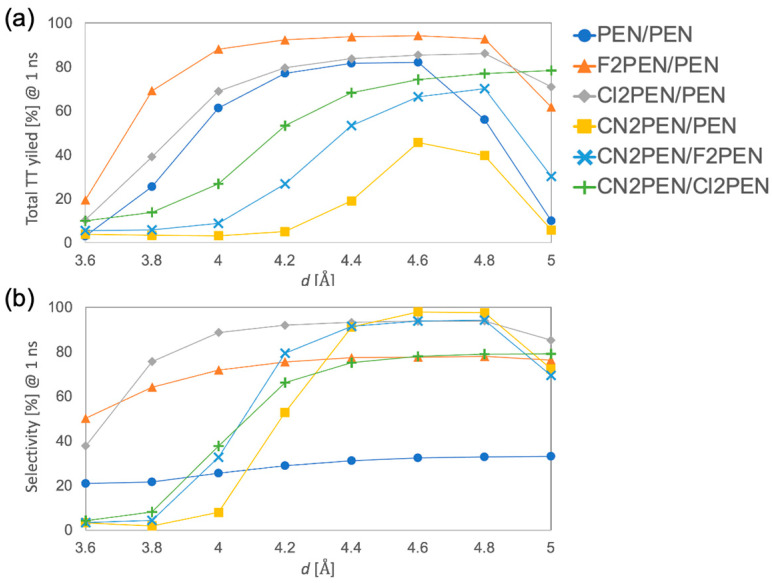
(**a**) The total TT yield and (**b**) the selectivity of the separated TT state at *t* = 1 ns.

Finally, SF dynamics simulations were performed by numerically solving the TCL-QME for the constructed exciton Hamiltonian. We conducted SF dynamics simulations for **PEN/PEN**, **F2PEN/PEN**, **Cl2PEN/PEN**, **CN2PEN/PEN**, **CN2PEN/F2PEN**, and **CN2PEN/Cl2PEN**. Figure 8a,b shows the results of total TT yield (denoted as *y*) and selectivity (denoted as *s*) of the separated TT pair state (T_1_S_0_T_1_) as a function of *d* [Å] at *t* = 1 ns. Note that, in general, the spin-decoherence of the TT state is about ns to µs order according to the many time-resolved EPR studies. The data at *t* = 10 ps were presented in the Supplementary Materials. For small *d*, the TT populations usually converged to a specific value~10^1^ ps. However, for larger *d* (>4.8 Å), the TT population dynamics converged at the timescale of 1 ns, which is comparable to the timescale of the spin-decoherence process [14]. Thus, we should consider the spin-decoherence process to fully understand the SF dynamics of the systems with large *d*.

As seen in Figure 8a, the total TT yield at *d* = 3.6 Å was low (<20%) for all these systems, which is due to the strong interaction between S_1_-CT states. Except for **CN2PEN/Cl2PEN**, whose total TT yield continued to increase in the range up to *d* ≤ 5 Å, the TT yields of the other systems showed an initial increase and then a decreasing trend as the *d* increased. The range of *d* where the total TT yield increased depends on the system: the total TT yield surpassed 70% at *d* = 4.2–4.8 Å in **PEN/PEN**, at *d* = 4.0–4.8 Å in **F2PEN/PEN**, at *d* = 4.2–4.8 Å in **Cl2PEN/PEN**, at *d* = 4.8 Å in **CN2PEN/F2PEN**, and at *d* = 4.6–4.8 Å in **CN2PEN/PEN**. The TT yield of **CN2PEN/PEN** was low and took a local maximum (45%) at *d* = 4.6 Å. The reduction in TT yield at larger *d* (>4.8 Å) is attributed to the small π-orbital overlap between the neighboring molecules, leading to the slow SF rate with a time constant of >1 ns.

As shown in Figure 8b, the selectivity for **PEN/PEN** (homotrimer) showed slight *d*-dependence and low values (20–35%). In contrast, the selectivity increased gradually as the *d* increased from 3.6 Å to 4.8 Å for **F2PEN/PEN** and **Cl2PEN/PEN**. Furthermore, drastic enhancement of the selectivity was obtained for **CN2PEN/Y** (**Y** = **PEN**, **F2PEN**, **Cl2PEN**) in the range of *d* = 3.8–4.4 Å. It was improved to >70% at *d* = 4.0–4.8 Å in **F2PEN/PEN**, at *d* = 3.8–5.0 Å in **Cl2PEN/PEN**, at *d* = 4.4–5.0 Å in **CN2PEN/PEN**, at *d* = 4.2–4.8 Å in **CN2PEN/F2PEN**, and at *d* = 4.4–5.0 Å in **CN2PEN/Cl2PEN**. The optimal condition for balancing high *y* and high *s* is supposed to be at *d* = 4.8 Å in **Cl2PEN/PEN** (*y* = 86.1%, *s* = 93.8%, and *y* × *s* = 80.8%) at long timescale *t* = 1 ns, or *d* = 4.2 Å (*y* = 79.6%, *s* = 91.9%, and *y* × *s* = 73.2%) at short timescale *t* = 10 ps. These cases exhibited higher selectivity of separated TT states than **PEN/PEN** because of the lower T_1_ energy of ***X*** than ***Y***.

**Figure 9:**
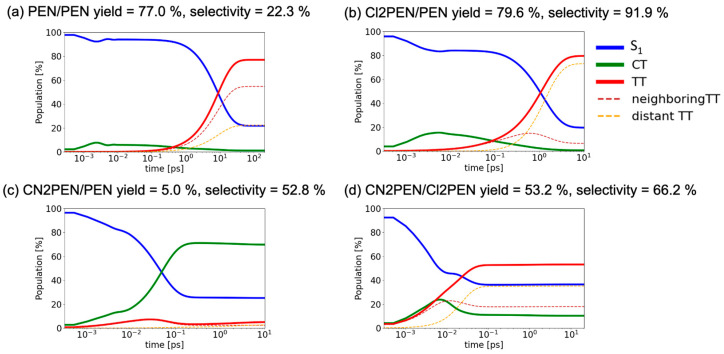
Time-evolution of the diabatic population of S_1_, CT, and TT states for **PEN/PEN**, **Cl2PEN/PEN**, **CN2PEN/PEN**, and **CN2PEN/Cl2PEN** systems with intermolecular distance *d* = 4.2 Å.

Figure 9 shows the time-evolutions of the diabatic state populations for (a) **PEN/PEN** (*y* = 77.0% and *s* = 22.3%), (b) **Cl2PEN/PEN** (*y* = 79.6% and *s* = 91.9%), (c) **CN2PEN/PEN** (*y* = 5.0% and *s* = 52.8%), and (d) **CN2PEN/Cl2PEN** (*y* = 53.2% and *s* = 66.2%) at *d* = 4.2 Å. The horizontal axis is given on a log scale to see the slow increase in the TT population at large. *d*. From Figure 9a, the total TT population of **PEN/PEN** increased at ~10^1^ ps, which consisted of both the neighboring and distant TT contributions. The selectivity of the distant TT state was lower than that of neighboring TT in this homotrimer model. For **Cl2PEN/PEN** [Figure 9b], the total TT population increased at ~10^0^ ps, about one order faster than **PEN/PEN**. In this system, the distant TT population was more significant than the neighboring TT population in the range of 10^0^–10^1^ ps. For **CN2PEN/PEN** [Figure 9c], the TT population remained low whereas the CT population became high in the time scale of 10^−2^–10^−1^ ps. This is because the C*^Y^*A*^X^* state is lower than the S_1_ and TT states, as mentioned in Figure 8a. For **CN2PEN/Cl2PEN** [Figure 9d], the TT generation occurred at ~10^−2^ ps, which is about 10^3^ times faster than that in **PEN/PEN**. Although the converged value of the total TT population was ~50%, the high selectivity of the distant TT was achieved. Next, we discuss the origin of faster SF in heterotrimers **Cl2PEN/PEN** and **CN2PEN/Cl2PEN** than in homotrimer **PEN/PEN** in view of quantum interference.

## 4. Discussion

**Figure 10:**
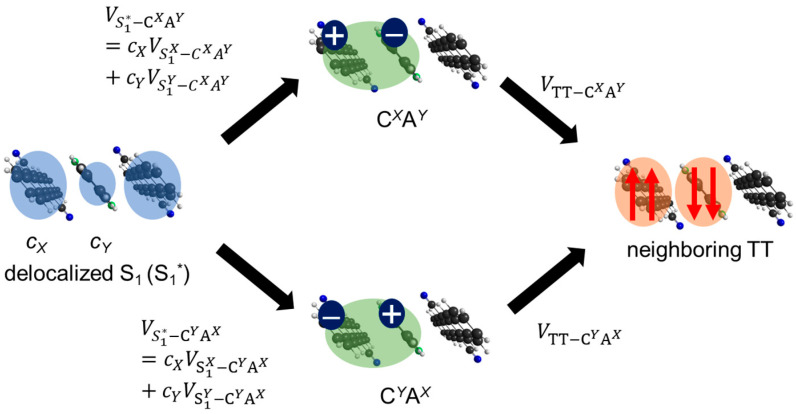
Schematic illustration of the cancellation mechanism of the effective S_1_-TT mixing between C*^X^*A*^Y^*-mediate and C*^Y^*A*^X^*-mediate interaction paths.

The origin of the faster SF in heterotrimers is attributed to the effects of quantum interference due to the stabilization of CT states. In a previous study [24], we demonstrated how the stabilization of one CT configuration (CA or AC) by local structural symmetry breaking resolved the quantum interference between the interaction paths via the CA and AC states, which caused the cancellation in S_1_-TT mixing in pentacene ring-shaped aggregate models. According to this study, the SF tends to occur faster when the adiabatic S_1_-like state includes the TT wavefunction component or the adiabatic TT-like state includes the S_1_-wavefunction component. However, in some highly symmetric aggregate structures, such as slip-stack dimers or ring-shaped aggregates, the S_1_-TT mixing vanishes because, as shown in Figure 10, two effective interaction paths between delocalized S_1_ state (denoted as S_1_* state) and TT state mediated by C*^X^*A*^Y^* and C*^Y^*A*^X^* state cancel each other. For simplicity, let us consider a hetero-dimer system consisting of ***X*** and ***Y***. By applying the second-order perturbation theory, the correlation between the delocalized S_1_* and TT is estimated by the following equation:

(8) $$V^{\prime }=\sqrt{\frac{3}{2}}\left(\frac{V_{LH}}{E_{S_{1}^{*}}-E_{TT}}\frac{c_{X}V_{LL}-c_{Y}V_{HH}}{E_{S_{1}^{*}}-E_{C^{X}A^{Y}}}+\frac{V_{HL}}{E_{S_{1}^{*}}-E_{TT}}\frac{c_{Y}V_{LL}-c_{X}V_{HH}}{E_{S_{1}^{*}}-E_{C^{Y}A^{X}}}\right)$$

Here, the S_1_* is described as the linear combination of the $S_{1}^{X}$ and $S_{1}^{Y}$, and its expansion coefficients are represented by $c_{X}$ and $c_{Y}$, respectively. The first and the second terms in the right-hand side of Equation (8) correspond to the upper and the lower CT-mediated interaction paths between S_1_* and TT states provided in Figure 10, respectively. For pentacene homodimer (***X*** = ***Y***) with H-aggregate type structure, inserting the relations $c_{X}=-c_{Y}=1/\sqrt{2}$, $V_{LH}=V_{HL}$, and $E_{C^{X}A^{Y}}=E_{C^{Y}A^{X}}$, the right-hand side of Equation (8) becomes zero. The same situation can be obtained for the symmetric J-aggregate-type structure where $c_{X}=c_{Y}=1/\sqrt{2}$, $V_{LH}=-V_{HL}$, and $E_{C^{X}A^{Y}}=E_{C^{Y}A^{X}}$. However, in hetero-dimer cases (***X*** ≠ ***Y***), the energy of C*^Y^*A*^X^* is more stabilized than C*^X^*A*^Y^*, and the second term in the right-hand side of Equation (8) becomes larger than the first term, resulting in the larger S_1_-TT mixing. The correlation between the S_1_ and TT states becomes large when the energy gap between S_1_, TT, and C*^Y^*A*^X^* states (the denominators of the right-hand side of Equation (8)) is small. However, the population of the CT state becomes large when CT state energy is too low, as shown in the SF dynamics results of **CN2PEN/PEN**.

**Figure 11:**
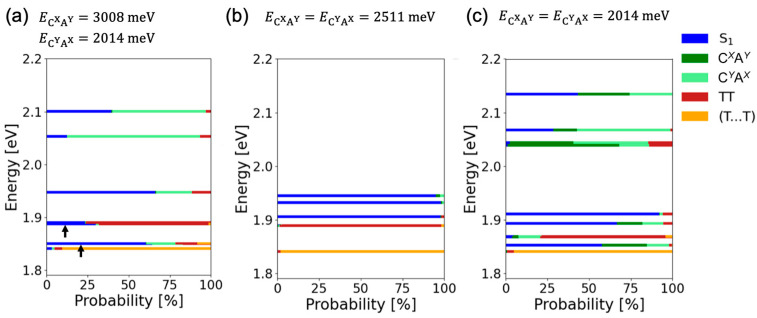
(**a**) Calculation results of adiabatic state energies and percentages of the diabatic states in **CN2PEN/Cl2PEN** at *d* = 4.2 Å, and results when the CT state energies are parametrically set to (**b**) the average of C*^Y^*A*^X^* and C*^X^*A*^Y^* state energies, and (**c**) the C*^Y^*A*^X^* state energy. The C*^X^*A*^Y^*-like adiabatic state energies are too high in case (**b**) to be presented within the energy range of 1.8–2.2 eV. The adiabatic states pointed by the black arrows in (**a**) indicate the S_1_-TT mixed states.

Figure 11a shows the calculation results of adiabatic state energies and percentages of the diabatic states in **CN2PEN/Cl2PEN** at *d* = 4.2 Å. To see how the quantum interference affects the energies and percentages of the diabatic states, we also evaluated these quantities by replacing the CT state energies in the exciton Hamiltonian with the average of C*^Y^*A*^X^* and C*^X^*A*^Y^* state energies (Figure 11b) and with the C*^Y^*A*^X^* state energy (Figure 11c), while other electronic coupling parameters remained unchanged. For pristine **CN2PEN/Cl2PEN**, considerable S_1_-TT hybridization occurs in the S_1_-like adiabatic states with an energy of ~1.85 eV and in the TT-like adiabatic states with an energy of ~1.9 eV. However, the S_1_-TT mixing was small for the latter cases with symmetrized coupling parameters. These results demonstrated how the stabilization of one CT state promoted the S_1_-TT mixing and enhanced the rate of SF.

## 5. Conclusions

This study presents an efficient strategy for controlling the spatiotemporal evolution of TT pairs generated by the SF in heterotrimers, ***X/Y***. We performed TDDFT calculations for pre-screening based on effective energy-matching conditions for hetero-fission and SF dynamics simulations based on the TCL-QME approach combined with the exciton Hamiltonian construction at the XMC-QDPT2 to find optimal conditions for balancing high TT yield and high selectivity of the separated TT. Considering the quantum interference, the energies and wavefunctions of adiabatic exciton states were analyzed to clarify the roles of asymmetric CT states in an efficient hetero-fission process. There are two advantageous points to consider when considering such a hetero-fission system.

First, we can expect directional TT migration from the center to the terminal molecules, with a probability of over 70%. Directional TT migration efficiencies correlate with $\left|\sigma_{p}\right|$ of substituents. Introducing halogen atoms or CN groups with large $\left|\sigma_{p}\right|$ into pentacene lowers the S_1_ and T_1_ excitation energies. Thus, we can tune the T_1_ energy difference between ***X*** and ***Y*** by choosing the appropriate species of ***X*** and ***Y***, resulting in the higher selectivity of spatially separated TT states.

Second, we can expect an acceleration of TT generation owing to the asymmetric energy levels of the CT states. When the $\sigma_{p}$ of the substituents is larger in ***X*** than ***Y***, the C*^Y^*A*^X^* state is energetically more stable than the C*^X^*A*^Y^* state. Such a reduction in symmetry breaks the destructive quantum interference between the S_1_-TT mixing paths mediated by C*^Y^*A*^X^* and C*^X^*A*^Y^* states. The degree of energy stabilization of C*^Y^*A*^X^* states is determined by the intermolecular distance and the electron-withdrawing ability of substituents, while the off-diagonal exciton Hamiltonian elements are almost unchanged. It is clarified that there is an optimal difference in $\sigma_{p}$ for substituents in ***X*** and ***Y*** to enhance SF efficiency.

We expect that the results and conclusions based on the present model apply to the cases of 2D and 3D organic frameworks consisting of different molecular species or intramolecular SF for systems of covalently linked multimers in realistic situations. The present results suggested that an optimal region of intermolecular distance to achieve efficient TT separation was relatively large (>4 Å) compared with the usual distances between the neighboring π-planes in actual molecular crystals. In addition, we should consider the effects of the polar environment, such as the crystal field or solvation field, since they often contribute to the stabilization of CT energy [20,21]. In addition, it is necessary to consider the populations of high-spin states in the TT dissociation process to establish valuable quantum spin technology strategies [15,26,27,28]. Nevertheless, the present study is expected to open a new way to enhance SF by utilizing the hetero-fission process.

## Data Availability

Data are contained within the article or Supplementary Material.

## Supplementary Materials

The following supporting information can be downloaded at https://www.mdpi.com/article/10.3390/molecules29225449/s1, Figure S1: diabatic energy of ***X*/*Y*** with *d* ranging from 3.6 to 5.0 Å; Figure S2: diabatic coupling of ***X/Y*** with *d* ranging from 3.6 to 5.0 Å; Figure S3: TT population and distant TT selectivity at time 10 ps.; Table S1: all electronic coupling parameter of ***X/Y***.; Table S2: TT yield *y* [%] and distant TT selectivity *s* [%] for ***X/Y***.

## Appendix A

**Table A1:** S_1_ and T_1_ excitation energies, ST gaps, HOMO, and LUMO energies, and HL gaps evaluated by the (TD)DFT calculations, and *para*-substitution coefficients for pentacene derivatives, σ_p_. Energies are given in eV.

Molecule	*E*(S_1_)	*E*(T_1_)	ST-Gap	HOMO	LUMO	HL-Gap	σ_p_^*1^
**PEN**	2.335	1.127	1.2079	−5.89	−1.56	4.33	0
**F2PEN**	2.261	1.070	1.1916	−5.95	−1.69	4.26	0.06
**Cl2PEN**	2.220	1.035	1.1845	−6.11	−1.91	4.20	0.23
**CN2PEN**	2.067	0.935	1.1324	−6.59	−2.59	4.01	0.66
**CF_3_2PEN**	2.188	1.015	1.1728	−6.40	−2.20	4.20	0.54
**Pr2PEN ^*2^**	2.226	1.054	1.1725	−5.73	−1.51	4.21	−0.13
**OCH_3_2PEN**	2.212	1.041	1.1711	−5.74	−1.53	4.21	−0.27
**Ar2PEN ^*2^**	2.259	1.085	1.1749	−5.77	−1.51	4.27	−0.01
**Thi2PEN ^*2^**	2.235	1.065	1.1702	−5.90	−1.67	4.23	0.05

^*1^ The data are obtained from ref. [52]. ^*2^ **Pr2PEN**: R = C_3_H_7_, **Ar2PEN**: R = C_6_H_5_, **Thi2PEN**: R = C_4_H_3_S.
