# Supplementary material for: Theoretical Study on Singlet Fission Dynamics and Triplet Migration Process in Symmetric Heterotrimer Models
Source: Molecules. 2024 Nov 19;29(22):5449. doi: 10.3390/molecules29225449 (PMC11597243; doi:10.3390/molecules29225449)
Supplement: Supplementary file 1 [file molecules-29-05449-s001.zip › molecules-3306963-supplementary.pdf]

Supporting Information for

"Theoretical Study on Singlet Fission Dynamics and Triplet Migration Process in Symmetric Heterotrimer Models"

Hajime Miyamoto 1, Kenji Okada 1, Kohei Tada 1,2,3, Ryohei Kishi 1,2,3,4,* and Yasutaka Kitagawa 1,2,3,4,5

1 Graduate School of Engineering Science, Osaka University, Toyonaka 560-8531, Osaka, Japan
2 Research Center for Solar Energy Chemistry (RCSEC), Graduate School of Engineering Science, Osaka University, Toyonaka 560-8531, Osaka, Japan
3 Center for Quantum Information and Quantum Biology (QIQB), Osaka University, Toyonaka 560-8531, Osaka, Japan
4 Innovative Catalysis Science Division, Institute for Open and Transdisciplinary Research Initiatives (ICS-OTRI), Osaka University, Suita 565-0871, Osaka, Japan
5 Spintronics Research Network Division, Institute for Open and Transdisciplinary Research Initiatives (OTRI-Spin), Toyonaka 560-8531, Osaka, Japan
* Correspondence: kishi.ryohei.es@osaka-u.ac.jp

Contents

I. Details for the calculation of XMC-QDPT2

II. Electronic coupling parameters

III. TT population and distant TT selectivity at t = 10 ps

I. Details for calculation of XMC-QDPT2/6-31G(d)

In this section, we show detailed information for the calculation of diabatic coupling at the XMC-QDPT2 level.[1,2] Before the XMC-QDPT2 calculation, we optimized pentacene monomer structure at the CAM-B3LYP/6-31G(d) level using Gaussian16. Then, we conducted state-averaged (SA)-CASSCF(2,2)/6-31G(d) calculation to obtain the reference orbitals for the molecular highest occupied molecular orbitals (HOMOs) and the lowest unoccupied molecular orbitals (LUMOs) using GAMESS-US program package. The state average was taken for the lowest four states with equivalent weights specifying PURES = .F.. Then, SA-CASSCF(4,4)/6-31G(d) calculation for dimer structure was conducted to obtain four diabatic MOs (DMOs) localized to reference molecular HOMOs and LUMOs by using Nakamura-Truhlar's 4-fold way scheme in both singlet and triplet states. The DMOs are used for the XMC-QDPT2/6-31G(d) calculations of singlet and triplet excited states, respectively, to obtain the eigen energies and eigenvectors for all the excited state in dimer systems. The intruder state avoidance parameter in the XMC-QDPT2 calculation was set to 0.02. In calculation with CAS (4,4) model, such as CASSCF (4,4) and XMC-QDPT2, the state average was taken as the equivalent average across all states (twenty states for singlets and fifteen states for triplets).

The adiabatic energy $\{\mathcal{E}_a\}$ and adiabatic wavefunction $|\Phi_a\rangle$ is obtained as the linear combination of diabatic CSFs $|\Psi_m\rangle$ as follows:

$$|\Phi_a\rangle = \sum_m C_{ma}\,|\Psi_m\rangle \tag{S1}$$

where the $\{C_{ma}\}$ is the expansion coefficients. The diabatic state energy and couplings parameters related to SF were obtained from the adiabatic state energy $\{\mathcal{E}_a\}$ and their configuration interaction coefficients $\{C_{ma}\}$ by the following equations:

$$\begin{aligned} E_m = \langle\Psi_m|H|\Psi_m\rangle &= \sum_{a,b}\langle\Psi_m|\Phi_a\rangle\langle\Phi_a|H|\Phi_b\rangle\langle\Phi_b|\Psi_m\rangle \\ &= \sum_{a,b} C_{ma}C_{mb}\mathcal{E}_b\delta_{ab} = \sum_a |C_{ma}|^2\mathcal{E}_a \end{aligned} \tag{S2}$$

$$\begin{aligned} V_{mn} = \langle\Psi_m|H|\Psi_n\rangle &= \sum_{a,b}\langle\Psi_m|\Phi_a\rangle\langle\Phi_a|H|\Phi_b\rangle\langle\Phi_b|\Psi_n\rangle \\ &= \sum_{a,b} C_{ma}C_{nb}\mathcal{E}_b\delta_{ab} = \sum_a C_{ma}C_{nb}\mathcal{E}_a \end{aligned} \tag{S3}$$

For triplet-triplet exciton transfer coupling, the direct coupling and its correction due to perturbative ^{3}CT-mediated transfer path was calculated by eq. (7) with parameters obtained from triplet-state calculation.

In the calculation of diabatic state energy in triplet excited states, we can obtain the T_1 energy difference between molecule X and Y ($\Delta E_{\mathrm{T}} = E(T_1^X) - E(T_1^Y)$). In other words, the TT state energy of $\mathrm{T}^X\mathrm{T}^X$ is approximately obtained from $E(\mathrm{T}_1^X\mathrm{T}_1^Y)$ and ΔE_{T} as $E(\mathrm{T}_1^X\mathrm{T}_1^X) = E(\mathrm{T}_1^X\mathrm{T}_1^Y) + \Delta E_{\mathrm{T}}$ without calculating XMC-QDPT2 in trimer model.

II. Electronic coupling parameters

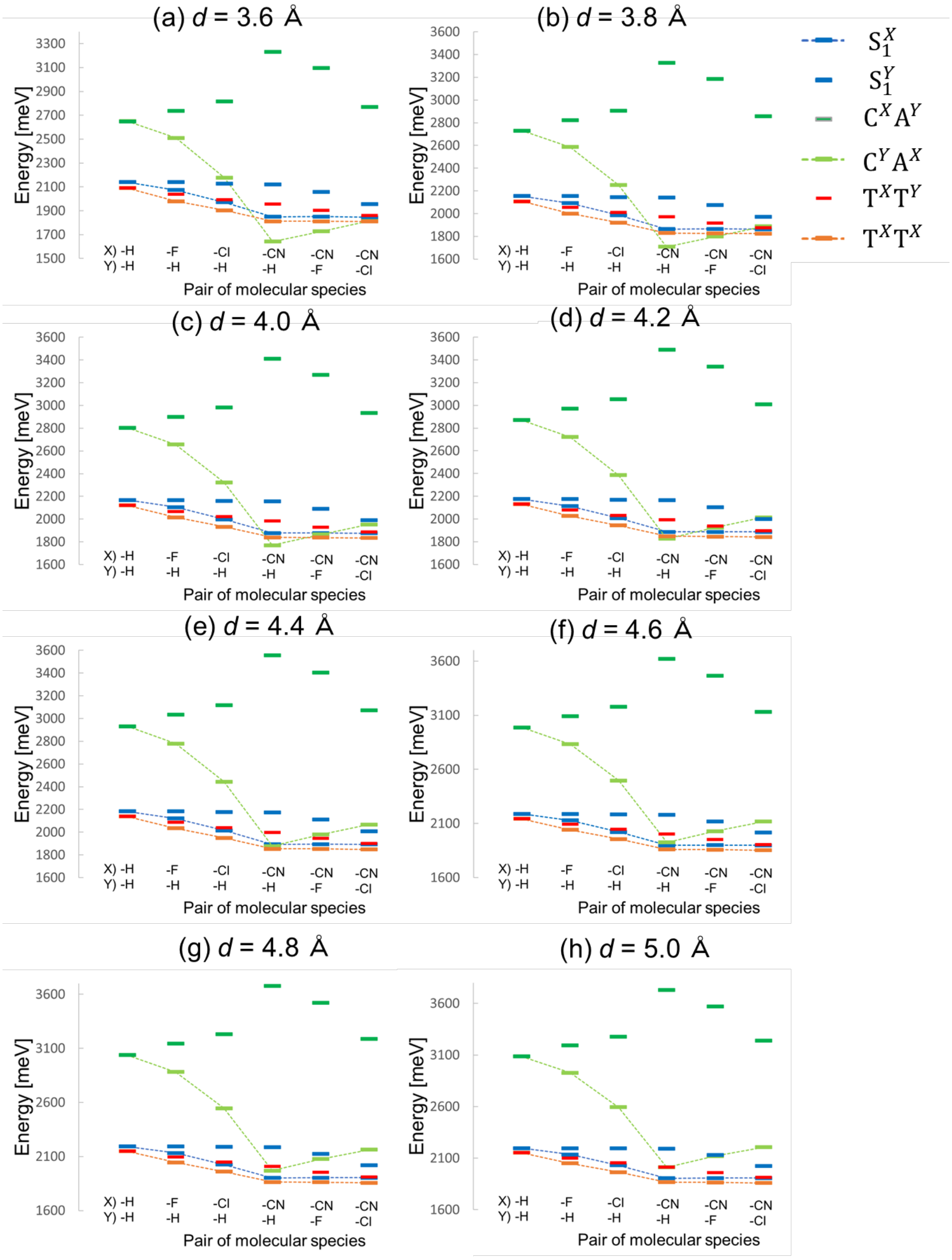

Figure S1. Diabatic energy of ***X*/*Y*** with *d* ranging from 3.6 to 5.0 Å

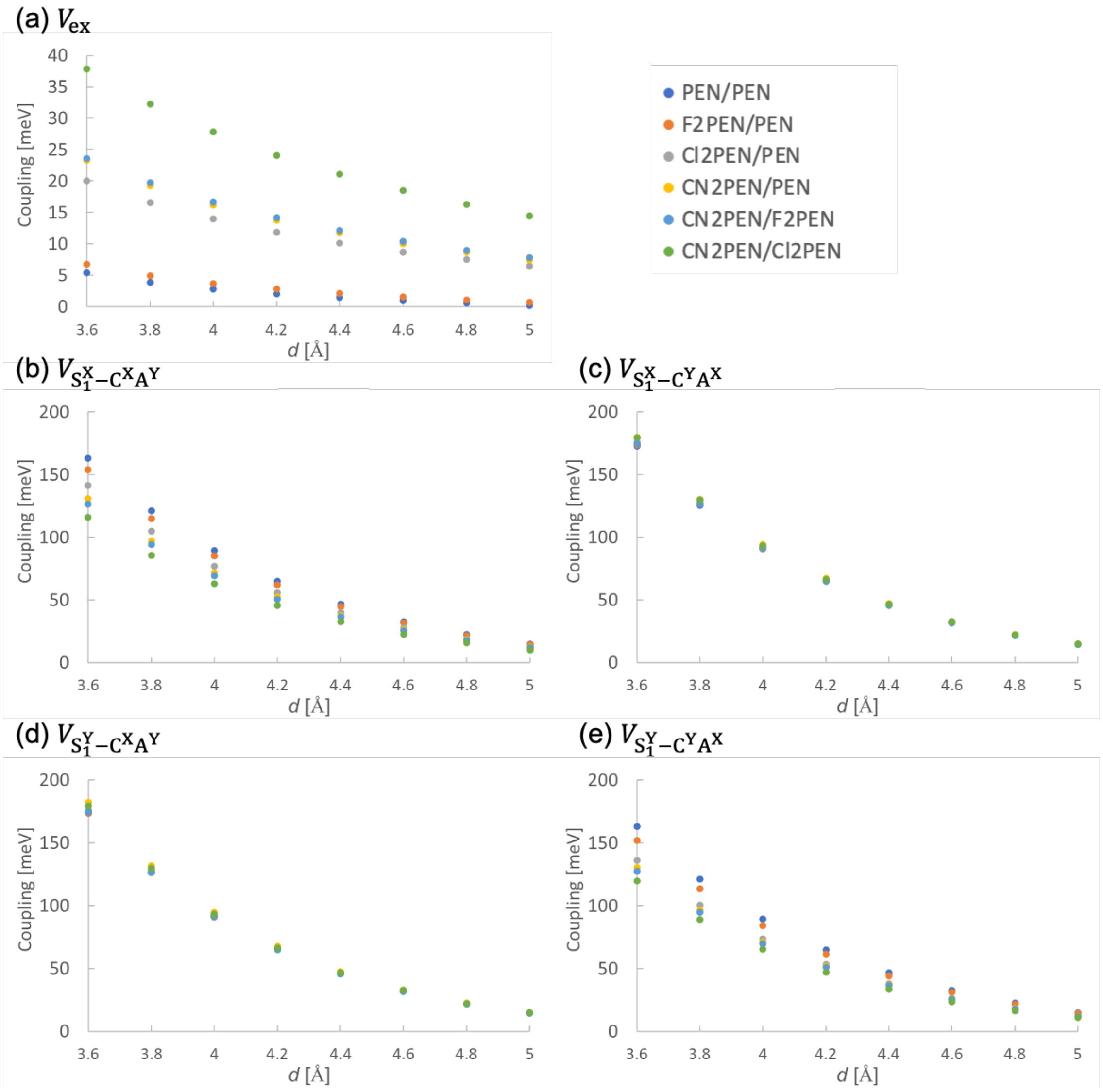

(a) V_{ex}
Coupling [meV]
40
35
30
25
20
15
10
5
0
3.6
3.8
4
4.2
4.4
4.6
4.8
5
d [Å]
PEN/PEN
F2PEN/PEN
Cl2PEN/PEN
CN2PEN/PEN
CN2PEN/F2PEN
CN2PEN/Cl2PEN
(b) $V_{S_1^X-C^XA^Y}$
(c) $V_{S_1^X-C^YA^X}$
(d) $V_{S_1^Y-C^XA^Y}$
(e) $V_{S_1^Y-C^YA^X}$
Coupling [meV]
200
150
100
50
0
3.6
3.8
4
4.2
4.4
4.6
4.8
5
d [Å]

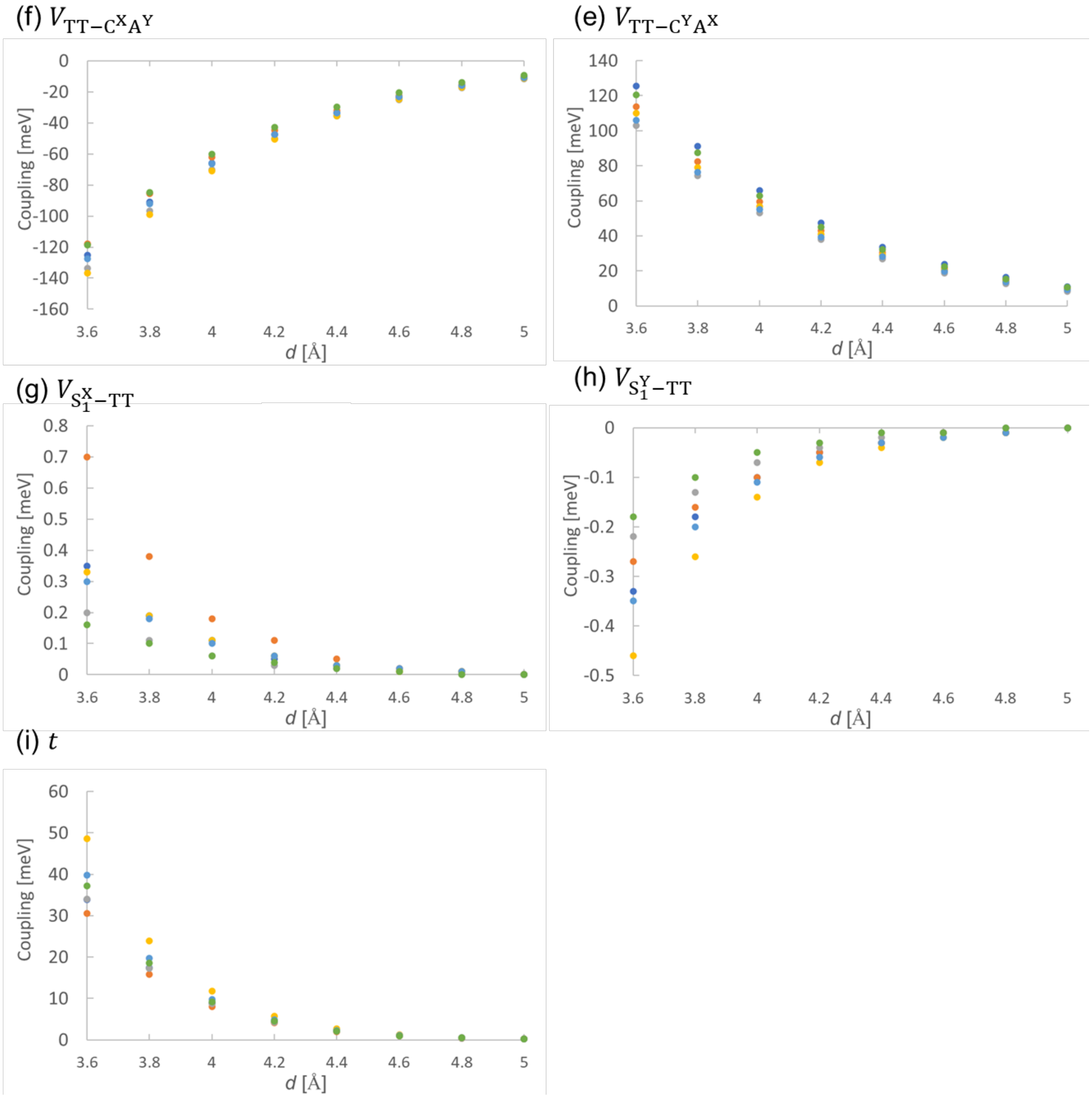

Figure S2. Diabatic coupling of ***X/Y*** with *d* ranging from 3.6 to 5.0 Å

Table S1. All electronic coupling parameter of ***X/Y***.

(a) PEN/PEN

d [Å]	V_{ex}	$V_{S_1^X-C^XA^Y}$	$V_{S_1^X-C^YA^X}$	$V_{S_1^Y-C^XA^Y}$	$V_{S_1^Y-C^YA^X}$	$V_{TT-C^XA^Y}$	$V_{TT-C^YA^X}$	$V_{S_1^X-TT}$	$V_{S_1^Y-TT}$	t
3.6	5.43	162.95	172.83	173.47	162.95	-125.29	125.39	0.35	-0.33	33.86
3.8	3.82	121.33	125.64	126.33	121.46	-91.08	91.18	0.19	-0.18	17.46
4	2.76	89.47	90.73	91.21	89.55	-65.92	65.98	0.11	-0.1	8.9
4.2	2.01	65.11	64.9	65.23	65.16	-47.37	47.4	0.05	-0.05	4.47
4.4	1.42	46.62	45.85	46.09	46.65	-33.68	33.7	0.03	-0.03	2.19
4.6	0.95	32.77	31.88	32.04	32.76	-23.58	23.64	0.01	-0.01	1.04
4.8	0.55	22.5	21.77	21.89	22.49	-16.24	16.25	0.01	-0.01	0.48
5	0.22	15.08	14.53	14.62	15.07	-10.94	10.94	0	0	0.21

(b) F2PEN/PEN

d [Å]	V_{ex}	$V_{S_1^X-C^XA^Y}$	$V_{S_1^X-C^YA^X}$	$V_{S_1^Y-C^XA^Y}$	$V_{S_1^Y-C^YA^X}$	$V_{TT-C^XA^Y}$	$V_{TT-C^YA^X}$	$V_{S_1^X-TT}$	$V_{S_1^Y-TT}$	t
3.6	6.71	153.8	161.76	173.55	151.89	-117.69	113.71	0.7	-0.27	30.61
3.8	4.91	114.87	117.34	126.48	113.48	-85.65	82.31	0.38	-0.16	15.82
4	3.69	84.99	85.06	90.68	84	-62.17	59.46	0.18	-0.1	8.1
4.2	2.81	62.09	60.07	65.88	61.41	-44.68	42.84	0.11	-0.05	4.08
4.4	2.12	44.64	42.75	46.12	44.2	-31.88	30.56	0.05	-0.03	2.01
4.6	1.55	31.48	29.78	32.04	31.19	-22.4	21.53	0.02	-0.01	0.96
4.8	1.08	21.71	20.38	21.84	21.53	-15.38	14.9	0.01	-0.01	0.44
5	0.69	14.6	13.64	14.56	14.49	-10.39	10.1	0	0	0.2

(c) Cl2PEN/PEN

d [Å]	V_{ex}	$V_{S_1^X-C^XA^Y}$	$V_{S_1^X-C^YA^X}$	$V_{S_1^Y-C^XA^Y}$	$V_{S_1^Y-C^YA^X}$	$V_{TT-C^XA^Y}$	$V_{TT-C^YA^X}$	$V_{S_1^X-TT}$	$V_{S_1^Y-TT}$	t
3.6	20.03	141.6	175.77	175.95	136.32	-133.48	103.05	0.2	-0.22	34.05
3.8	16.61	104.96	127.76	127.92	100.74	-96.86	74.23	0.11	-0.13	17.29
4	13.97	77.02	92.17	92.29	73.72	-69.97	53.24	0.06	-0.07	8.7
4.2	11.87	55.78	65.88	65.95	53.29	-50.2	37.86	0.03	-0.04	4.31
4.4	10.14	39.74	46.53	46.56	37.89	-35.67	26.69	0.02	-0.02	2.09
4.6	8.7	27.75	32.38	32.38	26.4	-25	18.6	0.01	-0.01	0.98
4.8	7.49	18.95	22	22.38	18.02	-17.2	12.65	0	0	0.44
5	6.48	12.62	14.87	14.57	11.98	-11.61	8.43	0	0	0.19

(d) CN2PEN/PEN

d [Å]	V_{ex}	$V_{\mathrm{S}_1^X-\mathrm{C}^X\mathrm{A}^Y}$	$V_{\mathrm{S}_1^X-\mathrm{C}^Y\mathrm{A}^X}$	$V_{\mathrm{S}_1^Y-\mathrm{C}^X\mathrm{A}^Y}$	$V_{\mathrm{S}_1^Y-\mathrm{C}^Y\mathrm{A}^X}$	$V_{\mathrm{TT}-\mathrm{C}^X\mathrm{A}^Y}$	$V_{\mathrm{TT}-\mathrm{C}^Y\mathrm{A}^X}$	$V_{\mathrm{S}_1^X-\mathrm{TT}}$	$V_{\mathrm{S}_1^Y-\mathrm{TT}}$	t
3.6	23.29	130.95	179.85	182.27	130.25	-136.84	110.21	0.33	-0.46	48.64
3.8	19.31	97.36	130.37	131.47	96.7	-98.83	79.11	0.19	-0.26	23.93
4	16.22	71.69	93.92	94.64	71.13	-71	56.77	0.11	-0.14	11.75
4.2	13.75	52.18	66.98	67.5	51.73	-50.57	40.88	0.06	-0.07	5.71
4.4	11.73	37.4	47.19	47.59	37.07	-35.65	29.17	0.03	-0.04	2.73
4.6	10.04	26.35	32.72	32.96	26.11	-24.77	20.47	0.01	-0.02	1.27
4.8	8.63	18.17	22.26	22.31	18.01	-16.89	14.14	0.01	-0.01	0.57
5	7.45	12.25	14.81	14.86	12.14	-11.27	9.56	0	0	0.25

(e) CN2PEN/F2PEN

d [Å]	V_{ex}	$V_{\mathrm{S}_1^X-\mathrm{C}^X\mathrm{A}^Y}$	$V_{\mathrm{S}_1^X-\mathrm{C}^Y\mathrm{A}^X}$	$V_{\mathrm{S}_1^Y-\mathrm{C}^X\mathrm{A}^Y}$	$V_{\mathrm{S}_1^Y-\mathrm{C}^Y\mathrm{A}^X}$	$V_{\mathrm{TT}-\mathrm{C}^X\mathrm{A}^Y}$	$V_{\mathrm{TT}-\mathrm{C}^Y\mathrm{A}^X}$	$V_{\mathrm{S}_1^X-\mathrm{TT}}$	$V_{\mathrm{S}_1^Y-\mathrm{TT}}$	t
3.6	23.63	126.49	172.86	174.34	127.7	-127.55	105.88	0.3	-0.35	39.73
3.8	19.71	94.07	125.49	126.53	94.76	-92.02	76.48	0.18	-0.2	19.76
4	16.63	69.38	90.38	91.09	69.74	-66.11	55.09	0.1	-0.11	9.78
4.2	14.15	50.57	64.49	64.92	50.74	-47.23	39.44	0.06	-0.06	4.79
4.4	12.11	36.34	45.46	45.69	36.41	-33.4	28.02	0.03	-0.03	2.31
4.6	10.41	25.66	31.55	31.72	25.68	-23.29	19.68	0.02	-0.02	1.08
4.8	8.97	17.75	21.48	21.61	17.75	-15.94	13.6	0.01	-0.01	0.49
5	7.77	11.98	14.31	14.4	11.98	-10.67	9.22	0	0	0.21

(f) CN2PEN/Cl2PEN

d [Å]	V_{ex}	$V_{\mathrm{S}_1^X-\mathrm{C}^X\mathrm{A}^Y}$	$V_{\mathrm{S}_1^X-\mathrm{C}^Y\mathrm{A}^X}$	$V_{\mathrm{S}_1^Y-\mathrm{C}^X\mathrm{A}^Y}$	$V_{\mathrm{S}_1^Y-\mathrm{C}^Y\mathrm{A}^X}$	$V_{\mathrm{TT}-\mathrm{C}^X\mathrm{A}^Y}$	$V_{\mathrm{TT}-\mathrm{C}^Y\mathrm{A}^X}$	$V_{\mathrm{S}_1^X-\mathrm{TT}}$	$V_{\mathrm{S}_1^Y-\mathrm{TT}}$	t
3.6	37.82	115.73	177.86	179.3	119.77	-118.51	120.6	0.16	-0.18	37.27
3.8	32.29	85.7	128.67	129.71	88.84	-84.66	87.37	0.1	-0.1	18.57
4	27.8	62.8	92.45	93.21	65.22	-60.3	62.97	0.06	-0.05	9.19
4.2	24.12	45.48	65.81	66.39	47.31	-42.67	45.13	0.04	-0.03	4.5
4.4	21.05	32.44	46.3	46.73	33.81	-29.89	32.07	0.02	-0.01	2.16
4.6	18.47	22.47	32.08	32.41	23.72	-20.64	22.54	0.01	-0.01	1.01
4.8	16.29	15.59	21.83	22.06	16.31	-13.98	15.46	0	0	0.46
5	14.44	10.14	14.53	14.7	10.95	-9.27	10.54	0	0	0.2

III. TT population and distant TT selectivity at *t* = 10 ps

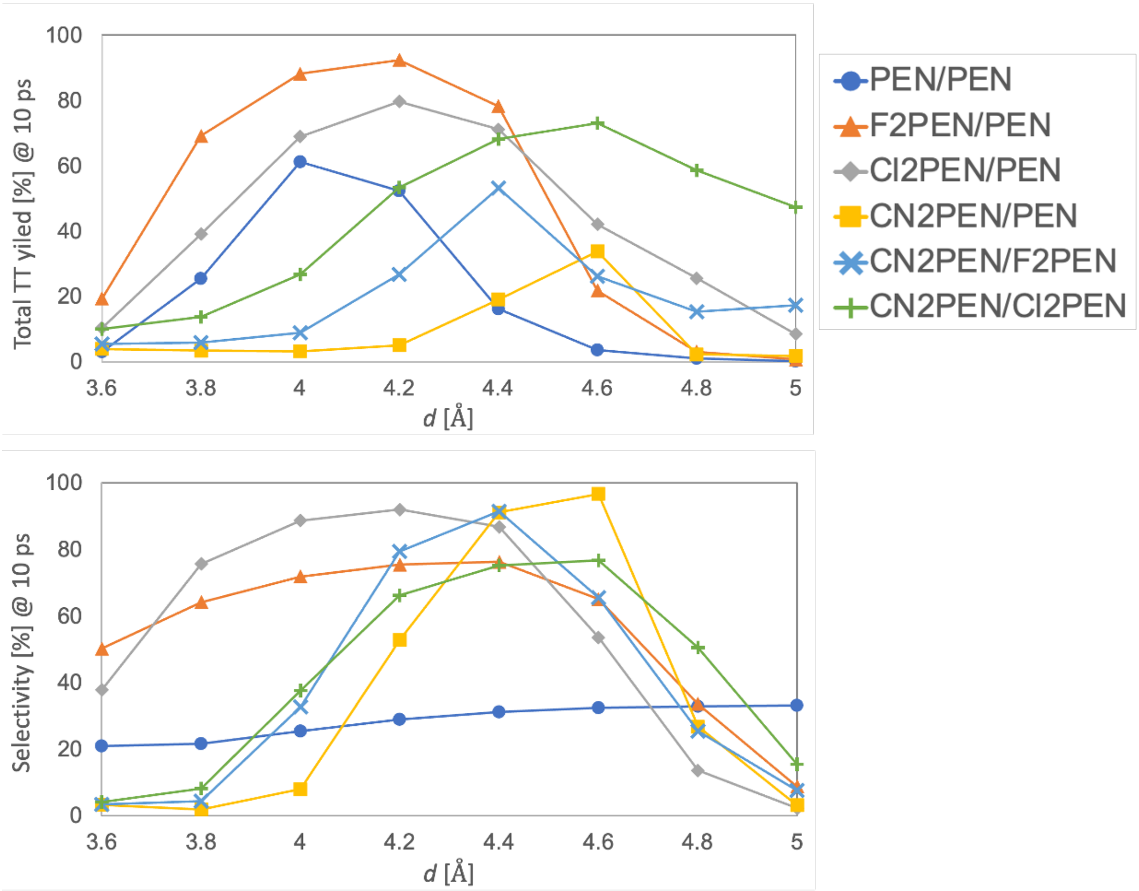

Figure S3. TT population and distant TT selectivity at time 10 ps.

Table S2. TT yield *y* [%] and distant TT selectivity *s* [%] for ***X/Y***.

X	*Y*	*d* [Å]	*t* = 10 ps		t = 1 ns	
			y [%]	*s* [%]	*y* [%]	*s* [%]
**PEN**	**PEN**	3.6	2.9	20.9	2.9	20.9
**PEN**	**PEN**	3.8	25.4	21.6	25.4	21.6
**PEN**	**PEN**	4	61.2	25.4	61.2	25.4
**PEN**	**PEN**	4.2	52.3	28.9	77	28.9
**PEN**	**PEN**	4.4	16.1	31.1	81.6	31.1
**PEN**	**PEN**	4.6	3.5	32.3	82.1	32.3
**PEN**	**PEN**	4.8	0.9	32.8	55.9	32.8
**PEN**	**PEN**	5	0.1	33.1	9.9	33.1
**F2PEN**	**PEN**	3.6	19.3	50.1	19.3	50.1
**F2PEN**	**PEN**	3.8	69.1	64.1	69.1	64.1
**F2PEN**	**PEN**	4	88.1	71.8	88.1	71.8
**F2PEN**	**PEN**	4.2	92.3	75.4	92.3	75.4
**F2PEN**	**PEN**	4.4	78.2	76.2	93.7	77.3
**F2PEN**	**PEN**	4.6	21.7	65	94.2	77.5
**F2PEN**	**PEN**	4.8	3	33.5	92.7	77.9
**F2PEN**	**PEN**	5	0.6	8.7	61.7	76.3
**Cl2PEN**	**PEN**	3.6	10.3	37.7	10.3	37.7
**Cl2PEN**	**PEN**	3.8	39	75.6	39	75.6
**Cl2PEN**	**PEN**	4	68.9	88.6	68.9	88.6
**Cl2PEN**	**PEN**	4.2	79.6	91.9	79.6	91.9
**Cl2PEN**	**PEN**	4.4	71.1	86.7	83.7	93.2
**Cl2PEN**	**PEN**	4.6	42	53.5	85.4	93.7
**Cl2PEN**	**PEN**	4.8	25.5	13.5	86.1	93.8
**Cl2PEN**	**PEN**	5	8.4	2.2	70.9	85.2
**CN2PEN**	**PEN**	3.6	3.8	3.2	3.8	3.2
**CN2PEN**	**PEN**	3.8	3.3	1.8	3.3	1.8
**CN2PEN**	**PEN**	4	3.1	7.9	3.1	7.9
**CN2PEN**	**PEN**	4.2	5	52.7	5	52.8
**CN2PEN**	**PEN**	4.4	19	91.1	19	91.1

**CN2PEN**	**PEN**	4.6	33.7	96.6	45.6	97.9
**CN2PEN**	**PEN**	4.8	2.2	26.7	39.6	97.5
**CN2PEN**	**PEN**	5	1.7	3.1	5.7	72.3
**CN2PEN**	**F2PEN**	3.6	5.4	3.3	5.4	3.3
**CN2PEN**	**F2PEN**	3.8	5.8	4.3	5.8	4.3
**CN2PEN**	**F2PEN**	4	8.8	32.6	8.8	32.6
**CN2PEN**	**F2PEN**	4.2	26.7	79.3	26.7	79.3
**CN2PEN**	**F2PEN**	4.4	53.1	91.4	53.2	91.4
**CN2PEN**	**F2PEN**	4.6	26.1	65.4	66.3	93.7
**CN2PEN**	**F2PEN**	4.8	15.2	25.4	70.1	94.2
**CN2PEN**	**F2PEN**	5	17.3	7.6	30.1	69.3
**CN2PEN**	**Cl2PEN**	3.6	9.9	4.1	9.9	4.1
**CN2PEN**	**Cl2PEN**	3.8	13.7	8.1	13.7	8.1
**CN2PEN**	**Cl2PEN**	4	26.7	37.6	26.7	37.6
**CN2PEN**	**Cl2PEN**	4.2	53.2	66.2	53.2	66.2
**CN2PEN**	**Cl2PEN**	4.4	68.2	75.1	68.2	75.1
**CN2PEN**	**Cl2PEN**	4.6	73	76.6	74.2	77.9
**CN2PEN**	**Cl2PEN**	4.8	58.6	50.4	76.9	78.9
**CN2PEN**	**Cl2PEN**	5	47.2	15.4	78.3	79
